# Supplementary material for: Pairing mechanism in the ferromagnetic superconductor UCoGe
Source: Nat Commun. 2017 Feb 23;8:14480. doi: 10.1038/ncomms14480 (PMC5473642; doi:10.1038/ncomms14480)
Supplement: Supplementary Information — Supplementary Figures 1-5, Supplementary Notes 1-3 and Supplementary References [file ncomms14480-s1.pdf]

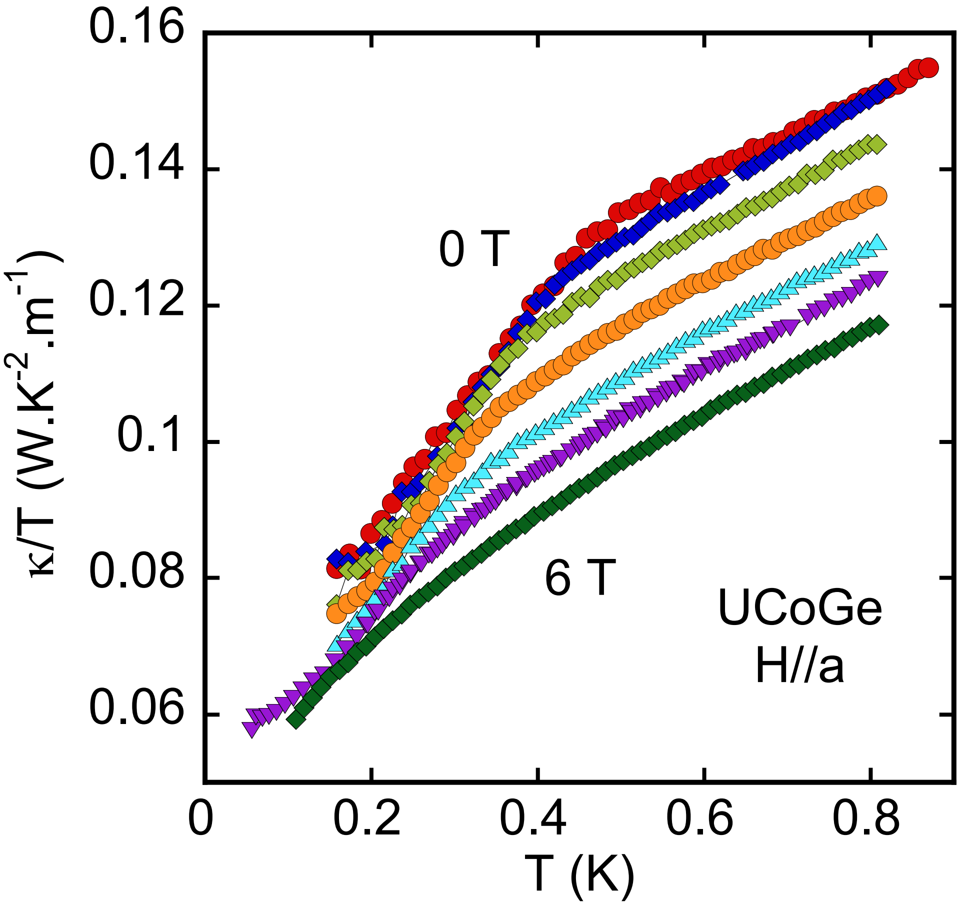

Supplementary Figure 1 | Raw data of thermal conductivity in UCoGe.

Some of the thermal conductivity measurements in UCoGe for magnetic fields along its **a**-axis.

From top to bottom : Every tesla from $\mu_0 H = 0$ T (red circles) to 6 T (dark green diamonds).

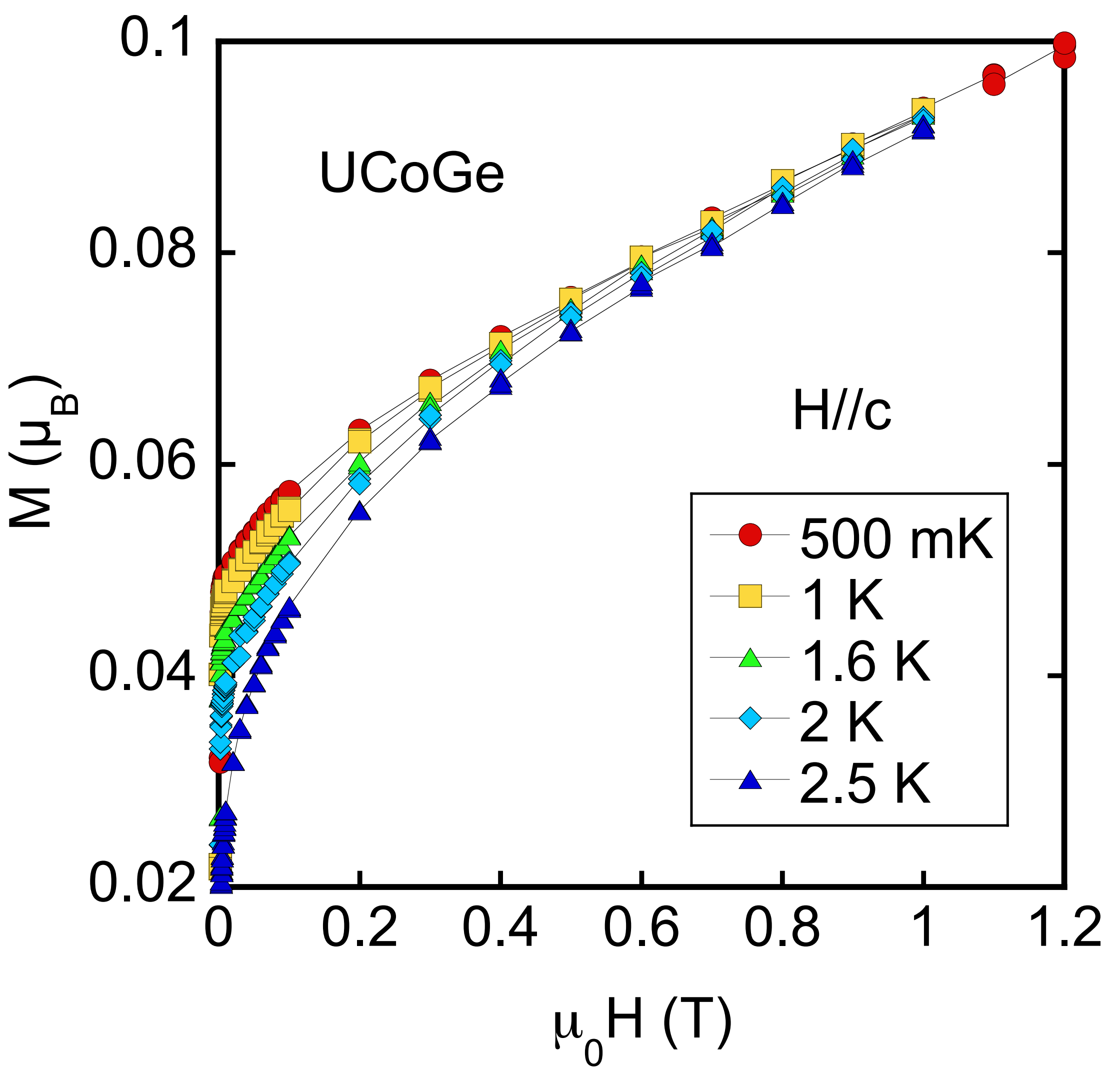

Supplementary Figure 2 | Low temperature magnetization of UCoGe.

Field dependence of the magnetization in UCoGe, for **H//c**, and at different temperatures, measured on the same sample used for the thermal conductivity measurements of H_{c2}. Red circles: $T = 500$ mK; yellow squares: $T = 1$ K; green triangles: $T = 1.6$ K; azure diamonds: $T = 2$ K and blue triangles: $T = 2.5$ K.

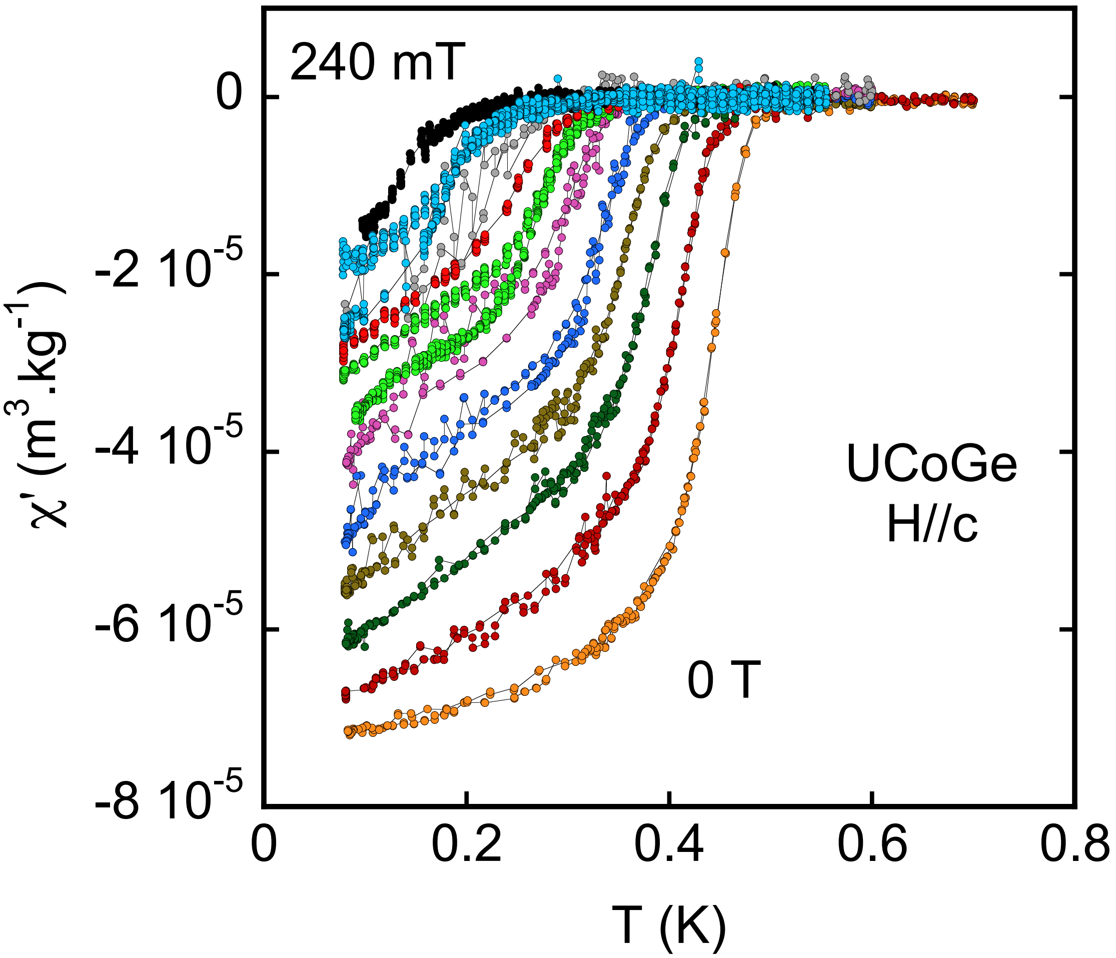

Supplementary Figure 3 | Superconducting transition in ac susceptibility.

The temperature dependence of ac-susceptibility in UCoGe, for different fields between 0 and 0.24 T along the **c**-axis. With increasing magnetic field, an hysteresis appears between up or down temperature sweeps measurements. The superconducting transition temperature is given by the intersection of linear interpolations in both the superconducting phase and normal phase.

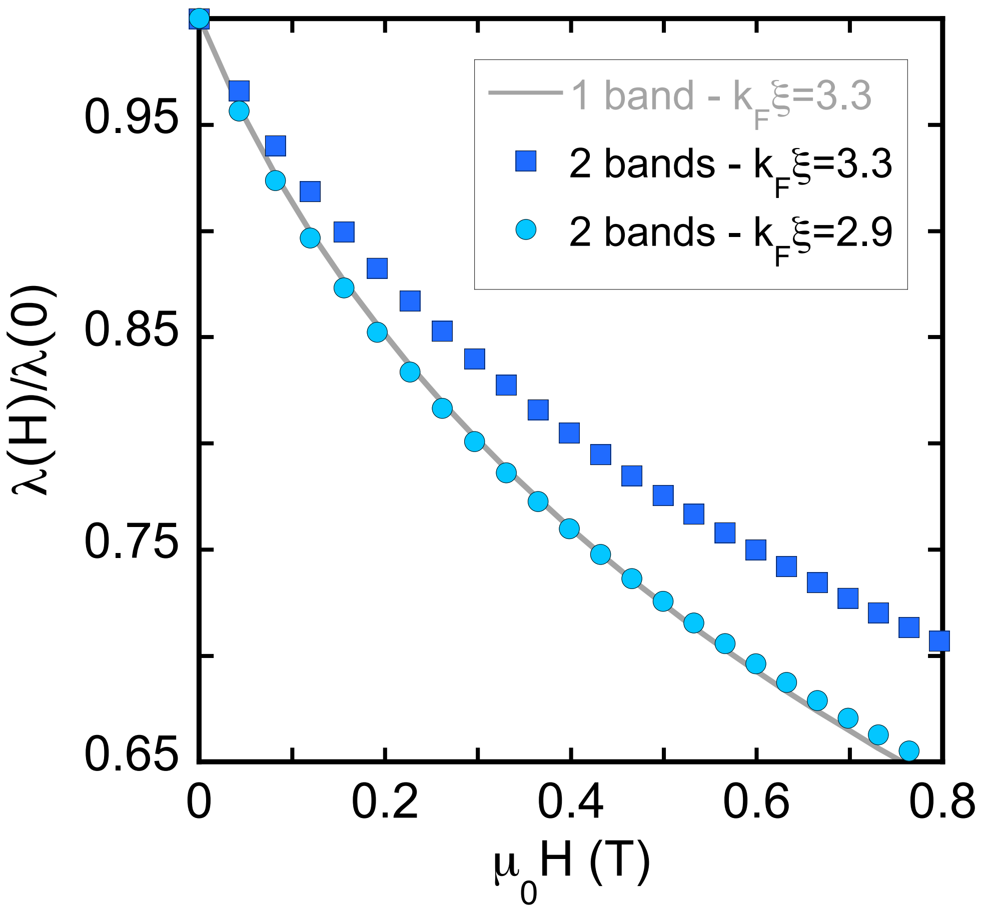

Supplementary Figure 4 | One vs two band calculations for UCoGe - H//c.

Grey full line: one band calculation of $\lambda(H)$ as presented in the main paper from the theoretical expressions in Ref.1. It is based on the experimental magnetization data (Fig.2), and a value of the the parameter $a = \xi_{\text{mag}} k_{\text{F}} \sim 3.3$. Dark blue squares: two-band calculation (from Ref.1), with the same magnetization data and same value of a, as well as with the experimental values of the susceptibility anisotropies between the **b-c** and **a-c** axis (see text). Light blue circle: same two-band calculation, but with a lower value of $a \sim 2.9$. One can see that the more exact two-band calculation yield the same field dependance as the one-band calculation, in the field range of $H_{c2}(0)$//**c**, with only a 10% adjustment of the value of a.

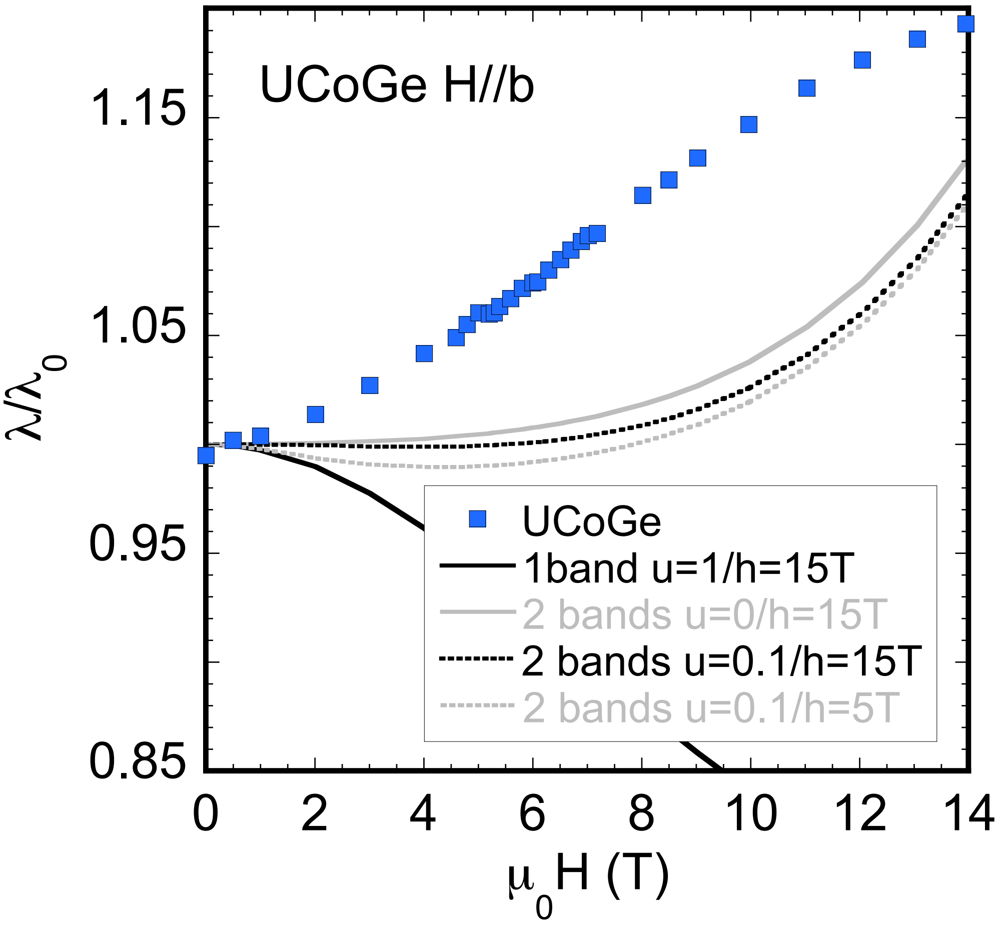

Supplementary Figure 5 | Comparison theory/experiment for H//b in UCoGe.

Blue squares: data for $\lambda(H)//\mathbf{b}$ as deduced from the H_{c2} data (see main paper). Full black line: one band calculation of $\lambda(H)$ according to Ref.1, taking now account of the rotation of the $\mathbf{d}$-vector, for a large exchange field of 15 T. λ should then decrease under field, as the rotation of $\mathbf{d}$ prevents to take full advantage of the fluctuations along the $\mathbf{c}$-axis. Full grey line: two-band calculation, with the same exchange field but still no polarization: this is the most favorable case, and an increase of λ is predicted for $\mathbf{H}//\mathbf{b}$, but the quantitative agreement is poor. Black dotted and grey lines: same two bands calculation, with 10% polarization of the bands, and respective exchange fields of 15 and 5 T. The agreement is even worse, and naturally, smaller exchange field accentuate the decrease of λ due to the rotation of the $\mathbf{d}$-vector.

Supplementary Note 1 | Two-band model in Mineev's theory:

In the work of V. Mineev[1,2], superconductivity is derived from a microscopic model, and ESP (Equal Spin Pairing) states are realized due to strong polarization of the Fermi sea in the exchange field driving the ferromagnetism. This naturally leads to a two-band solution for the superconductivity order parameter, where pairing is realized in the spin up or spin down bands. The splitting of the two bands can be characterized by a dimensionless parameter u, which corresponds to the relative change of the density of states ($N^{\uparrow,\downarrow}$) weighted by the particular choice of superconducting order parameter. For example, for $\Psi = (\hat{k_x}\eta_x^{\uparrow}, \hat{k_x}\eta_x^{\downarrow})$,

$$u = \frac{| < \hat{k_x}^2 N^{\uparrow}(k_x) > - < \hat{k_x}^2 N^{\downarrow}(k_x) > |}{< \hat{k_x}^2 N^{\uparrow}(k_x) > + < \hat{k_x}^2 N^{\downarrow}(k_x) >} \tag{1}$$

Eq.(6) presented in the main paper is a simplified version of these solutions, valid when the polarization is strong (u close to 1), or when the spin susceptibilities have a strong uniaxial anisotropy. This last point is well verified in UCoGe, which explains why we could safely ignore the complexity of the two-band solution. However, it is also easy to quantify numerically the importance of these corrections. The theoretical work of Ref.1 does not evaluate explicitely H_{c2}, which requires heavy calculations depending on the precise order parameter. But at the level discussed in our paper, it gives an expression for the coupling constant of ESP states (Eq.(117) in Ref.1) taking multiband effects into account. In all cases, T_{sc} is determined by a pairing constant λ:

$$\lambda = \frac{\lambda_{11} + \lambda_{22}}{2} + \sqrt{\frac{(\lambda_{11} - \lambda_{22})^2}{4} + \lambda_{12}\lambda_{21}} \tag{2}$$

The various λ_{ij} depend on the susceptibilities, but also on the band polarization (u), and on the orientation of the d-vector (representing the spin triplet superconducting order parameter): $(\mathbf{d} \times$

$\mathbf{d}^*$) is parallel to the average spin of the superconducting condensate, which should follow the effective field $\mathbf{H_m} = \mathbf{h} + \mathbf{H}$ with $\mathbf{h}$ the exchange field (//$\mathbf{c}$) and $\mathbf{H}$ the applied field. Noting ϕ the angle between $\mathbf{h}$ and $\mathbf{H}$ $\left(\tan\phi = \frac{H_y}{h}\right)$, χ_i the static susceptibility $\chi(\mathbf{H} = \mathbf{0}, \mathbf{q} = \mathbf{0})$ for the magnetization response along the field applied on axis-i, and taking also an isotropic $\mathbf{k}$-dependence for all susceptibilities (characterized by $a = \xi_{\text{mag}} k_{\text{F}}$), we get for the λ_{ij} from Eq.(117), (168)-(170) in Ref.1:

$$\lambda_{11} = \left[g_z \cos^2\phi + B\sin^2\phi\right](1+u) \quad ; \lambda_{22} = \left[g_z \cos^2\phi + B\sin^2\phi\right](1-u)$$
$$\lambda_{12} = \left[(A-g_z)\sin^2\phi + (A-B)\cos^2\phi\right](1-u) \quad ; \lambda_{21} = \left[(A-g_z)\sin^2\phi + (A-B)\cos^2\phi\right](1+u) \tag{3}$$

Combining Supplementary Eq.(3) with Supplementary Eq.(2), we have:

$$\lambda = \bar{\lambda}_0\left(g_z\cos^2\phi + B\sin^2\phi + \sqrt{u^2\left[g_z\cos^2\phi + B\sin^2\phi\right]^2 + (1-u^2)\left[(g_z-A)\sin^2\phi + (B-A)\cos^2\phi\right]^2}\right) \tag{4}$$

where

$$\bar{\lambda}_0 = \frac{\lambda(0)}{1+\sqrt{u^2+(1-u^2)(B-A)^2}} \tag{5}$$

and

$$g_z(\mathbf{H}) = \frac{\left(1+a^2\right)^2}{\left(\Theta(\mathbf{H})+a^2\right)^2}$$
$$\Theta(\mathbf{H}//\mathbf{c}) = 1/2\left(3\frac{M_z^2}{M_0^2} - 1\right); \quad \Theta(\mathbf{H}\perp\mathbf{c}) = \frac{T_{\text{Curie}}(H) - T_{\text{sc}}}{T_{\text{Curie}}(0) - T_{\text{sc}}} \tag{6}$$
$$A = \frac{\left(1+a^2\right)^2}{\left(\frac{\chi_z}{2\chi_x}+a^2\right)^2}; \qquad B = \frac{\left(1+a^2\right)^2}{\left(\frac{\chi_z}{2\chi_y}+a^2\right)^2}$$

Very generally, when there is no polarization ($u = 0$), Supplementary Eq.(4) is independent of the rotation of the $\mathbf{d}$-vector, and it reduces to:

$$\lambda = \lambda_0 \frac{g_z + B - A}{1 + B - A} \tag{7}$$

Supplementary Note 2 | Consequences for H//c:

For $\mathbf{H}$//$\mathbf{c}$, the $\mathbf{d}$-vector remains perpendicular to the $\mathbf{c}$-axis ($\phi = 0$). So Supplementary Eq.(4) takes a simple form, even with finite polarization u:

$$\lambda = \bar{\lambda}_0 \left(g_z + \sqrt{u^2 g_z^2 + (1 - u^2)(B - A)^2} \right) \tag{8}$$

The change in the field dependence of λ given by Supplementary Eq.(8), compared to the expression $\lambda = \lambda_0 g_z$ ($u = 1$) used for the discussion in the main paper, arises from the term $(B - A)$ which depends on the susceptibilities along $\mathbf{b}$ and $\mathbf{a}$ axis respectively, and is field independent. This term, which tends to diminish the field dependence of λ, will have the largest influence when there is no polarization ($u = 0$).

Collecting values of χ_a and χ_b from the literature[3–5], and our own data for $\chi_c \approx 0.11\ \mu_{\mathrm{B}}/\mathrm{T}$ at 0.5 K, we get the following estimations for the ratios $\chi_c/2\chi_b \sim 8.5$ and $\chi_c/2\chi_a \sim 20$. Supplementary Fig.4 displays the worst case, computed from Supplementary Eq.(7) ($u = 0$), when the influence of the two-band effect is the strongest. The difference between the one-band and the two-band calculation of $\lambda(H)$ is compensated, in the required field range (0-0.6 T), by a 10%

change of $\xi_{\text{mag}}k_{\text{F}}$. With a finite polarization u, the required change is even lower. This justifies the use the one-band approximation in the main paper for the whole discussion of H_{c2} in UCoGe along the $\mathbf{c}$-axis.

Supplementary Note 3 | Consequences for H//b:

As regards now the situation for field along the $\mathbf{b}$-axis, on top of the polarization, we also need the (related) value of the exchange field h, required to determine the rotation angle ϕ of the $\mathbf{d}$-vector. Starting with the one-band model ($u = 1$) with rotation of the $\mathbf{d}$-vector, Supplementary Fig.5 shows that Supplementary Eq.(4) predicts a decrease of λ for $\mathbf{H}$//$\mathbf{b}$. This counter-intuitive result is easily understood. In such a case, the expression for λ reduces to $\lambda(H)/\lambda(0) = g_z \cos^2\phi + B \sin^2\phi$, and the increase of g_z due to the suppression of T_{Curie} does not compensate the increasing weight of the fluctuations along $\mathbf{b}$ (B is much smaller than g_z since $\chi_b \ll \chi_c$) even with a large value of the exchange field (15 T chosen for the curve of Supplementary Fig.5).

The situation is better in a two-band model with negligible polarization (for the same 15 T exchange field, $u = 0$, see Supplementary Fig.5). In this case, λ has again the simple expression of Supplementary Eq.(7), independent of the angle ϕ, and it increases due to the suppression of T_{Curie}. The positive point is that one recovers an increase of λ along $\mathbf{b}$, however not the right value for the initial (positive) curvature, and more seriously, not the saturation of the increase at higher fields. This increase is controlled by the T_{Curie} suppression, and no change of the values

of the parameters can alter this behavior. Including the effect of a finite polarization only further increases this behavior. This is notably the case at low fields, where the trend to a decrease of λ is recovered (as for the one band case), the faster, the weaker the exchange field is (as it drives a faster rotation of the spins): see the calculations for 10% polarization and an exchange field of 15 or 5 T in Supplementary Fig.5. As a summary, for the case of $\mathbf{H}//\mathbf{b}$ in UCoGe, when a two-band model is considered (as it should), the theory of Ref.1 predicts indeed a pairing strength increasing with field, as is experimentally expected. But quantitative agreement of $\lambda(H)$ for this field direction is hard to obtain, featuring the complexity of magnetism in this case.

UCoGe is probably not the best system to discuss the physics at play for field along the $\mathbf{b}$-axis: the case of URhGe is much better documented experimentally, and an important information is provided again by very recent NMR experiments in (Co-doped) URhGe[6]. It has been observed that in this compound, both the longitudinal (T_1) and transverse (T_2) nuclear relaxation times, probing respectively the fluctuations transverse and along the effective $\mathbf{H_m}$ field, strongly increase when a field is applied along the $\mathbf{b}$-axis. This means that both fluctuations along $\mathbf{c}$ and along $\mathbf{b}$-axis are reinforced on field increase along $\mathbf{b}$. This last effect cannot be deduced from the model based on a Landau description of the ferromagnetic state[1,7], but could be captured in more microscopic models of the ferromagnetic state[8], where the reinforcement of superconductivity along the $\mathbf{b}$-axis originates from the appearance of soft magnon modes.

1. Mineev, V. P. Superconductivity in Uranium Ferromagnets. Preprint at https://arxiv.org/abs/1605.07319, accepted in *Physics Uspekhi (Advances in Physical Sciences)* (2016).

2. Mineev, V. P. Magnetic field dependence of pairing interaction in ferromagnetic superconductors with triplet pairing. *Phys. Rev. B* **83**, 064515 (2011).

3. Huy, N. T., de Nijs, D. E., Huang, Y. K. & de Visser, A. Unusual Upper Critical Field of the Ferromagnetic Superconductor UCoGe. *Phys. Rev. Lett.* **100**, 077002 (2008).

4. Hardy, F. *et al.* Transverse and longitudinal magnetic-field responses in the Ising ferromagnets URhGe, UCoGe, and UGe_2. *Phys. Rev. B* **83**, 195107 (2011).

5. Knafo, W. *et al.* High-field moment polarization in the ferromagnetic superconductor UCoGe. *Phys. Rev. B* **86**, 184416 (2012).

6. Tokunaga, Y. *et al.* Reentrant Superconductivity Driven by Quantum Tricritical Fluctuations in URhGe: Evidence from $^{59}\mathrm{Co}$ NMR in $\mathrm{URh_{0.9}Co_{0.1}Ge}$. *Phys. Rev. Lett.* **114**, 216401 (2015).

7. Mineev, V. P. Reentrant superconductivity in URhGe. *Phys. Rev. B* **91**, 014506 (2015).

8. Hattori, K. & Tsunetsugu, H. p-wave superconductivity near a transverse saturation field. *Phys. Rev. B* **87**, 064501 (2013).
